# Molybdenum Disulfide and Reduced Graphene Oxide Hybrids as Anodes for Low-Temperature Lithium- and Sodium-Ion Batteries

**DOI:** 10.3390/nano15110824

**Published:** 2025-05-29

**Authors:** Anna A. Vorfolomeeva, Alena A. Zaguzina, Evgeny A. Maksimovskiy, Artem V. Gusel’nikov, Pavel E. Plyusnin, Alexander V. Okotrub, Lyubov G. Bulusheva

**Affiliations:** Nikolaev Institute of Inorganic Chemistry SB RAS, 3 Acad. Lavrentiev Ave., 630090 Novosibirsk, Russia; vorfolomeeva@niic.nsc.ru (A.A.V.); kotsun@niic.nsc.ru (A.A.Z.); eugene@niic.nsc.ru (E.A.M.); gusel@niic.nsc.ru (A.V.G.); plus@niic.nsc.ru (P.E.P.); spectrum@niic.nsc.ru (A.V.O.)

**Keywords:** molybdenum disulfide, reduced graphene oxide, lithium-ion batteries, sodium-ion batteries, low temperature

## Abstract

Lithium- and sodium-ion batteries (LIBs and SIBs) suffer from the significant degradation of electrochemical performance at low temperatures. This work presents promising hybrid anodes synthesized by the rapid thermolysis of ammonium tetrathiomolybdate and graphene oxide (GO) at 600 and 700 °C. Transmission electron microscopy revealed the formation of MoS_2_ crystallites oriented along or perpendicular to the surface of reduced GO (rGO) layers. X-ray photoelectron spectroscopy found the covalent C–S bonds connecting components in the MoS_2_/rGO hybrids. The MoS_2_/rGO_600 hybrid showed higher specific capacities in LIBs of 1370 mAh/g, 835 mAh/g, and 711 mAh/g at a current density of 0.1 A/g and temperatures of 25 °C, 0 °C, and −20 °C, respectively, due to the presence of excess sulfur in the sample. Increasing the current density to 2 A/g retained 78 and 34% of the capacity at 25 °C and −20 °C. In SIBs, the MoS_2_/rGO_700 hybrid showed more promising results, achieving 550 mAh/g at 0.1 A/g and 400 mAh/g at 2 A/g, while lowering the temperature to −20 °C retained 48 and 17% of the capacity. Such good SIB performance is attributed to the enrichment of the sample with vertically oriented MoS_2_ layers covalently bonded to the rGO surface.

## 1. Introduction

Lithium-ion batteries (LIBs) have become widely used as energy sources and have found applications ranging from portable devices to electric transport [1,2]. The limited availability of lithium has prompted a search for alternative materials, and sodium is a promising candidate due to its abundance and low cost [3]. Sodium-ion batteries (SIBs) operate on the same principle as lithium-ion batteries, so similar materials can be used as components. Modern batteries must not only provide high energy and power density, be safe, charge quickly, and last for a long time, but also operate over a wide range of ambient temperatures. The degradation of LIBs and SIBs at low temperatures (LTs) is becoming increasingly problematic, preventing their wider use [4,5].

Metal-ion batteries perform best in the temperature range of 15–35 °C and lose capacity as the temperature decreases [6]. The traditional anode material used in LIBs remains graphite with a theoretical capacity of only 372 mAh/g [7]. At LTs, a number of problems arise, which can be summarized as follows: (1) the reduced ionic conductivity of the electrolyte and the solid electrolyte interphase (SEI) formed on the graphite surface [8,9]; (2) the limited diffusion of lithium ions in the graphite material [4,10]; (3) the high polarization of the graphite anode associated with the first two factors [5,11]; (4) significantly increased charge-transfer resistance at the electrolyte–electrode interfaces [12]. For SIBs, the search for anode material is an urgent problem, the solution of which, especially in the context of the above-mentioned problems, requires significant efforts.

LT conditions can present energetic barriers to the chemical reactions that occur during battery charging and discharging. In the field of LIBs, initial efforts were focused on finding suitable co-solvents in the electrolyte to lower its freezing point and form a more favorable SEI layer on the graphite electrode [13]. As a result, the kinetics of charge transfer at the electrode–electrolyte interface are improved [12,14,15]. However, some studies have shown that diffusion limitations within graphite are the main obstacles to battery performance [4,10]. Electrode development involves the targeted modification of the active materials of the cathode and anode to improve intercalation and de-intercalation processes at LTs.

Molybdenum disulfide (MoS_2_), with a large layer spacing of 0.62 nm and a high theoretical capacity value of 669 mAh/g, is a suitable anode material for both LIBs and SIBs [16,17]. The problems of poor cycling performance and low ionic conductivity of MoS_2_ are addressed by changing the structural characteristics and morphology [18], as well as by modifying the interface in the MoS_2_-based composite [19,20,21]. One of the successful solutions is the combination of nanostructured MoS_2_ and a conductive graphene component. For example, record values in LIBs were obtained for the 1T(octahedral)-MoS_2_ based electrode with Mo atoms confined in the “graphene nanoreactor” and which amounted to 1840 and 1157 mAh/g at a current density of 0.1 and 1 A/g, respectively [22]. Encouraging characteristics in SIBs were obtained for single-layered ultra-small MoS_2_ nanoplates embedded in carbon nanofibers, which amounted to 854 mAh/g at 0.1 A/g and 623 mAh/g at 1 A/g [23]. Despite the extensive study of MoS_2_-based materials such as LIB and SIB anodes at room temperature (RT) conditions, there are only three works devoted to the study of MoS_2_ or its hybrids with carbon at LTs. In work [24], the use of commercial µm-MoS_2_ allowed for achieving a specific capacity in the SIB of ~200 mAh/g at a current density of 0.05 A/g and a temperature of −30 °C. MoS_2_ nanosheets grown vertically on graphene sheets delivered 1077 mAh/g at 0.1 A/g at RT in LIBs and maintained ∼700 mAh/g even at −20 °C [25]. The MoS_2_/C hybrid synthesized by a hydrothermal method [26] showed a discharge capacity of 734.2 mAh/g at −20 °C at a current density of 0.1 A/g, which decreased to 140.9 mAh/g when the current density was increased to 3 A/g.

In this work, we focused on the LT performance of MoS_2_/carbon hybrid nanomaterials in both LIBs and SIBs. The nanomaterials were synthesized using an original synthesis procedure, namely, the rapid thermolysis of an aerogel containing ammonium tetrathiomolybdate (ATM, (NH_4_)_2_MoS_4_) and graphene oxide (GO). Placing this aerogel in a preheated reactor results in the formation of MoS_2_ nanoparticles and reduced GO (rGO) layers, and the high pressure generated by the released gases ensures a strong bonding between the components, as confirmed by X-ray photoelectron spectroscopy (XPS). The determining factor affecting the structure of the hybrid material in this process is the synthesis temperature and the component ratio. To increase the amount of redox active sites in the anode, the ratio of ATM to GO was chosen to theoretically obtain MoS_2_/rGO 3:1. Syntheses were carried out at 600 and 700 °C, and transmission electron microscopy (TEM) revealed that temperature affects the mutual orientation of the MoS_2_ and rGO layers. The electrochemical properties were studied using galvanostatic discharge–charge (GDC) cycling, cyclic voltammetry (CV), and electrochemical impedance spectroscopy (EIS) in the range from 25 to −20 °C. The obtained results show the great potential of MoS_2_/graphene hybrids for LT LIBs and SIBs.

## 2. Materials and Methods

### 2.1. Materials and Synthesis

ATM was synthesized by passing a hydrogen sulfide flow with a rate of 30 mL/s through an ammonium heptamolybdate tetrahydrate (NH_4_)_6_Mo_7_O_24_·4H_2_O solution for 4 h. Needle-shaped crystals of ATM formed at the base of the flask and were filtered off, washed with a cold ethanol solution, and dried in air. GO was synthesized by the modified Hummers’ method. The details of the synthesis and the results of the characterization of the product are described elsewhere [27].

The synthesis of MoS_2_/GO aerogels involved the dispersion of GO powder in distilled water by ultrasonic treatment, accompanied by parallel mechanical stirring. A freshly prepared aqueous solution of ATM was then added to the resulting suspension. The amounts of GO and ATM were calculated from the mass ratio of MoS_2_ to rGO 3:1 in the final product. The prepared suspension was then frozen in liquid nitrogen and subjected to lyophilic freeze-drying for a week. The obtained aerogels were subjected to rapid thermolysis in an inert atmosphere (Ar). In details, the vertical tubular quartz reactor was heated to the designated temperature (600 or 700 °C) and purged with high-purity Ar (99.998%, 5–7.5 mL/s,) for 10 min. Thereafter, the gas flow rate was reduced to 1 mL/s, and the aerogel was immersed in the quartz reactor for 15 s. Then, the reactor was removed from the furnace and naturally cooled to RT. The samples obtained represent gray flakes and are designated as MoS_2_/rGO_600 and MoS_2_/rGO_700. Control samples of rGO were prepared from GO aerogel at temperatures of 600 °C (rGO600) and 700 °C (rGO700) similarly to the procedure described above.

### 2.2. Instrumental Methods

X-ray diffraction (XRD) analysis was performed on a Shimadzu XRD-7000 (Shimadzu Europa GmbH, Duisburg, Germany) diffractometer at RT using Cu Kα radiation and a Ni filter on the reflected beam. Crystallographic parameters were taken from the JCPDS-PDF database. Raman spectra were recorded on a LabRAM HR Evolution (Horiba, Kyoto, Japan) spectrometer using an Ar^+^ laser line at 488 nm. Morphology of the samples was investigated by scanning electron microscopy (SEM) on a SEM 5000 microscope (SIQTEC, PRC, Hefei, China) at a 15 kV acceleration voltage. Elemental analysis was carried out by energy dispersive X-ray analysis (EDS) on Xplore 30 (Oxford Instruments, UK) detector. TEM images were obtained on a JEM−2200FS (JEOL Ltd., Tokyo, Japan) at an accelerating voltage of 200 kV. The samples for the TEM study were prepared by ultrasonic dispersing in ethanol and the consequent deposition of the suspension upon a “holey” carbon film supported on a copper grid.

The specific surface area of MoS_2_/rGO_600 and MoS_2_/rGO_700 was determined using low-temperature N_2_ sorption. Before measurements, the samples were degassed in a flow of N_2_ at 100 °C for about 2 h. Isotherms were obtained on a Sorbi MS analyzer (META, Novosibirsk, Russia) at −196 °C and used to calculate the SSA values by the Brunauer–Emmett–Teller (BET) method.

Thermogravimetric (TG) analysis, differential scanning calorimetry (DSC), and differential thermogravimetric (DTG) analysis were carried out in synthetic air with a heating rate of 10 °C/min. The measurements were performed on a NETZSCH STA 449F1 Jupiter instrument (Selb/Bayern, Germany). The sample was placed in an open Al_2_O_3_ crucible and heated from room temperature to 1000 °C. The processing of experimental data was carried out using a standard software package Proteus analysis (v.6.1.0. 2013).

XPS experiments were carried out on a FlexPS spectrometer equipped with a Phoibos 150 analyzer (SPECS Surface Nano Analysis GmbH, Berlin, Germany). The spectra were recorded using a monochromatic Al Kα (1486.71 eV) source. The analyzer transmission energy was 20 eV. The analytical chamber was evacuated to ~10^–10^ mbar. The processing of the spectra was carried out using Casa XPS software (version 2.3.15, Casa Software Ltd., Teignmouth, UK). after subtraction of the background signal by Shirley’s method. The sp^2^ C 1s peak at 284.6 eV was used to calibrate the binding energy. The fitting of the spectra was performed using symmetric lines as a product of the Gaussian and Lorentzian components, with the exception of the graphite component for the C 1s line where the Doniach–Sunjic function was used. The XPS S 2p spectra were fitted by spin–orbit doublets with a ratio of the components of 2:1 and separation of 1.18 eV. The XPS Mo 3d spectra were fitted by spin–orbit doublets with a ratio of the components of 3:2 and separation of 3.13 eV. The atomic concentrations of elements were calculated from the survey spectra taking into account the photoionization cross-sections.

### 2.3. Electrochemical Measurements

Electrochemical measurements of the LIBs and SIBs were carried out in a half-cell configuration using CR2032 coin cells. Hybrid materials were mixed with conductive additive (super P) and polyvinylidene fluoride (PVDF) at a weight ratio of 8:1:1, and N-methyl-2-pyrrolidone as solvent. The mixtures were thoroughly homogenized on a vortex. The resulting slurries were spread on copper foil substrates and dried in a vacuum oven at 70 °C for 12 h. The electrode area was 1.7 cm^2^, and the mass loading of active material was 0.5–0.7 mg. Cells were assembled in an Ar-filled glove box (O_2_ and H_2_O < 0.1 ppm). A piece of polypropylene or glass fiber separator was inserted between a lithium or sodium metal anode and a hybrid cathode. The electrolyte was a 1 M LiPF_6_ in a mixture of ethylene carbonate, dimethyl carbonate, and diethyl carbonate (EC:DMC:DEC, 1:1:1 by volume) with 5% fluoroethylene carbonate (FEC) as an additive for the LIBs and 1 M NaClO_4_ in a propylene carbonate (PC, 100% Vol) with 5% FEC as an additive for the SIBs.

GDC cycling was performed on NEWARE CT-3008 stations (Neware Technology Ltd., Shenzhen, China) at current densities in the range from 0.1 to 2 A/g and over the potential range of 0.01–2.50 V vs. Li/Li^+^ or Na/Na^+^. Ten operation cycles were performed at each current density of 0.1, 0.5, 1, 2, and again at 0.1 A/g. Low-temperature experiments were performed in a laboratory refrigerator equipped with a system of electrical contact and kept at constant temperatures of 0 °C or −20 °C (temperature measurement error is ±2 °C). A new series of batteries was collected for measurements at each temperature. The specific capacity values were calculated based on the mass of the active material (MoS_2_/rGO) in the electrode.

After 50 cycles of GDC at room temperature or at −20 °C, EIS measurements were carried out using a BCS-805 instrument (Biologic, Seyssinet-Pariset, France). EIS spectra were recorded at a cell potential of 2.5 V vs. Li/Li^+^ or Na/Na^+^ using an alternating voltage with an amplitude of 10 mV. The frequency was decreased from 10 kHz to 0.05 Hz. CV curves were measured for freshly assembled batteries after three GDC cycles in a potential range of 0.01–2.5 V vs. Li/Li^+^ or Na/Na^+^ at scan rates of 0.1, 0.3, 0.5, 0.7, and 1.0 mV/s. The contributions of diffusion and capacitive processes to the peak current were separated by the following equation: i(v) = k_1_v + k_2_v^1/2^, where i(v) is the total current, k_1_v is the current from a surface-controlled charge storage process (capacitive behavior), and k_2_v^1/2^ is the current from a diffusion-controlled process (diffusion behavior).

After completion of the electrochemical tests, the cells were opened in a glove box under argon atmosphere. The tested electrodes were washed with a large excess of diethyl carbonate in order to remove electrolytes and dried for 12 h. The resulting samples were used for ex situ studies.

## 3. Results

### 3.1. Characterization of Hybrid Materials

**Figure 1:**
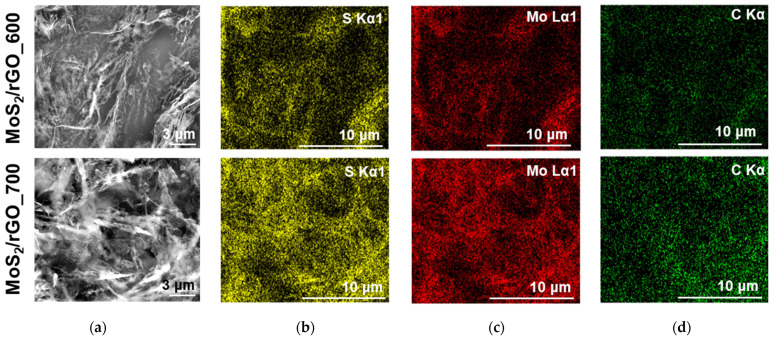
SEM images obtained in (**a**) the BSE mode and the corresponding elemental mapping with distribution of (**b**) sulfur (yellow), (**c**) molybdenum (red), and (**d**) carbon (green) for MoS_2_/rGO_600 (top) and MoS_2_/rGO_700 (bottom).

The hybrid materials were synthesized by rapidly heating the ATM/GO aerogel in an argon flow at temperatures of 600 and 700 °C. At these temperatures, GO loses oxygen groups and ATM decomposes to MoS_2_ [18]. Figure 1a presents the SEM images in the backscattered electron (BSE) mode for the MoS_2_/rGO_600 and MoS_2_/rGO_700 samples. The images show thin and wrinkled layers of exfoliated graphene and bright contrasting regions corresponding to MoS_2_. The elemental maps indicate a uniform distribution of Mo, S, and C in the samples (Figure 1b–d).

Low-resolution TEM images of MoS_2_/rGO_600 and MoS_2_/rGO_700 showed interconnected thin graphene layers coated with MoS_2_ (Figure S1). The flakes are crumpled, which is typical for graphene materials prepared by the rapid heating of GO-based aerogels [28]. The dark regions belong to MoS_2_, because molybdenum and sulfur are heavier than carbon. The uneven contrast in the graphene layers indicates the agglomeration of MoS_2_. Increasing the thermolysis temperature to 700 °C leads to the appearance of darker areas on the surface of the graphene flakes (Figure S1b).

**Figure 2:**
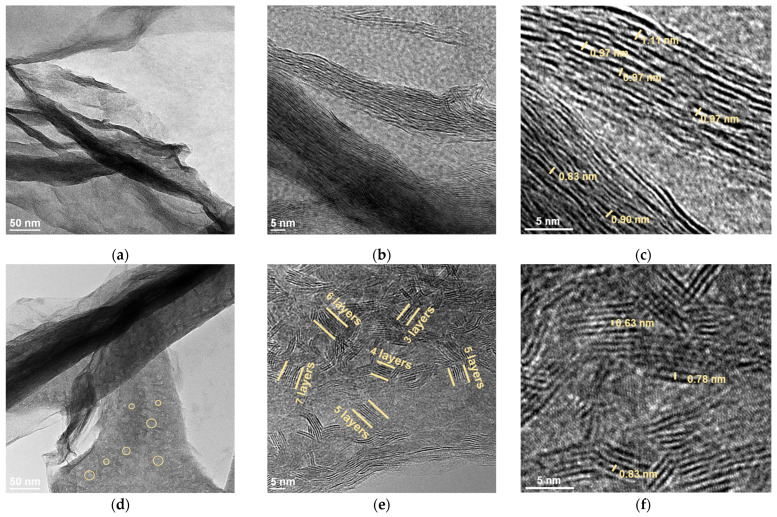
High-resolution TEM images of (**a**–**c**) MoS_2_/rGO_600 and (**d**–**f**) MoS_2_/rGO_700. The ovals in (**d**) show pores, the parallel lines in (**e**) indicate parallel layers of MoS_2_, and the numbers in (**c**,**f**) correspond to distances between the MoS_2_ layers.

Figure 2 compares TEM images of the samples obtained at higher magnifications. Transparent light gray areas indicate thin carbon layers; dark regions correspond to the MoS_2_ coating the rGO (Figure 2a,d). Most of the MoS_2_ layers are aligned with the rGO surface in MoS_2_/rGO_600 (Figure 2b). In some cases, the separation of the MoS_2_ layers is 0.83–1.11 nm (Figure 2c), which is significantly larger than the 0.62 nm value characteristics of the hexagonal MoS_2_ crystal. The increase in the distance may be due to the incorporation of graphene layers between the MoS_2_ layers. In the MoS_2_/rGO_700 sample, the stacks of MoS_2_ layers are oriented perpendicularly to the rGO surface (Figure 2e). The layers in the stacks are strongly curved, and the interweaving of layers from adjacent stacks forms pores (highlighted by yellow ovals in Figure 2d). The number of layers in the stacks varies from three to seven (Figure 2e), and the interplanar spacing is 0.62–0.83 nm (Figure 2f). The TEM study shows a change in the orientation of the thin MoS_2_ coating from parallel to perpendicular to the graphene layer at a temperature of 700 °C. A similar effect has previously been observed for MoS_2_ at temperatures of 800–850 °C [29,30].

The BET surface areas are 75 and 89 m^2^/g for MoS_2_/rGO_600 and MoS_2_/rGO_700, respectively, and are consistent with the value obtained for the MoS_2_-rGO composite with a similar component ratio [31].

**Figure 3:**
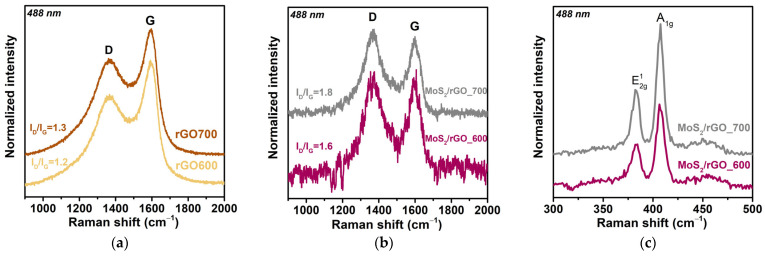
Raman spectra of (**a**) rGO samples obtained by GO exfoliation at 600 and 700 °C and (**b**,**c**) MoS_2_/rGO hybrids synthesized at 600 and 700 °C.

Raman spectra were analyzed to investigate the defect density of graphene sheets with and without MoS_2_. The spectra exhibit two broad peaks around 1368 cm^−1^ and 1597 cm^−1^, attributed to the D-band and G-band of rGO (Figure 3a,b). The D peak corresponds to the defects in the graphitic material, and the G peak is due to the in-plane vibrations of sp^2^-hybridized carbon. The ratio I_D_/I_G_ of the integral intensities of the D and G peaks was used to evaluate the defect density. The Raman spectra of pure rGO obtained by the thermal exfoliation of GO at 600 and 700 °C show similar defect densities of the samples (Figure 3a). In the hybrid materials, the I_D_/I_G_ value increases and is 1.6 for MoS_2_/rGO_600 and 1.8 for MoS_2_/rGO_700 (Figure 3b). This indicates the appearance of defects in the carbon matrix associated with the bonding of the rGO layers with MoS_2_.

In the lower wavenumber range (Figure 3c), the Raman spectra of the MoS_2_/rGO hybrids contain two peaks at 383.2 and 407.5 cm^−1^, corresponding to the E^1^_2g_ and A_1g_ modes, respectively [32]. The E^1^_2g_ mode is associated with in-plane vibrations, and the A_1g_ mode is due to the out-of-plane vibrations of S–Mo–S characteristic of hexagonal 2H-MoS_2_ [32]. The distance between the E^1^_2g_ and A_1g_ modes does not change with the increasing synthesis temperature and is 24.3 cm^−1^. This indicates that the average number of layers in the stacks is independent of the temperature used. The ratio of the A_1g_/E^1^_2g_ integral intensities is 1.3 and 1.5 for MoS_2_/rGO_600 and MoS_2_/rGO_700, respectively. A slight increase in the intensity of the A_1g_ mode in the MoS_2_/rGO_700 sample is due to the increase in vibrations perpendicular to the plane of the MoS_2_ layer and the suppression of in-plane vibrations [33]. This can be explained by the appearance of vertically oriented MoS_2_ layers bonded with graphene-like layers [34].

The XRD patterns of MoS_2_/rGO_600 and MoS_2_/rGO_700 exhibit peaks at ca. 2θ~14.0° (Figure S2a) corresponding to the (002) reflection of hexagonal 2H-MoS_2_. The position of the reflection is slightly shifted compared to the typical 2θ ~14.4° for MoS_2_ [35], indicating a slight increase in the interlayer spacing to 0.63 nm. The average number of layers in samples varies from four to five, which is consistent with the TEM data (Figure 2e). The presence of (hkl) reflections at ca. 33.2, 39.5, 50.1, and 59.1° in the XRD patterns of both samples characterizes the atomic ordering in the MoS_2_ layers. The peak appearing at 2θ ~ 24° is associated with the (002) graphite reflection of rGO. The low intensity and broadening of this reflection means that a small number of graphite layers are participating in the diffraction. The peak in the MoS_2_/rGO_700 pattern is weaker due to better exfoliation of the graphite sheets at a higher synthesis temperature.

TG analysis of MoS_2_/rGO_600 и MoS_2_/rGO_700 was carried out in air by heating the sample from RT to 1000 °C, as shown in Figure S3. The mass loss (~61.5 and ~52.2 wt%) that occurred from 216 to 500 °C is attributed to the conversion of MoS_2_ to MoO_3_ and the decomposition of rGO [36,37]. The MoS_2_ content is calculated to be about 68.4 and 58.1 wt% in the MoS_2_/rGO_600 и MoS_2_/rGO_700, respectively. From these data, the corresponding ratios of the MoS_2_ component to rGO are determined to be 2.2 and 1.4, respectively.

The survey XPS spectra exhibited signals of Mo, S, C, and O in the hybrid materials. The content of elements is presented in Table 1. The oxygen content does not exceed ~9 wt% in the samples and is associated with the oxidized states of sulfur, molybdenum, and oxygen-containing groups remaining in rGO. The presence of oxygen on the surface of the MoS_2_ nanoparticles is explained by the contact with air molecules. The mass ratio of the MoS_2_ component (sum of the Mo and S content) to carbon was found to be 1.7 for MoS_2_/rGO_600 and 1.5 for MoS_2_/rGO_700. A comparison of the TG and XPS data revealed that, in the MoS_2_/rGO_700 sample, MoS_2_ is localized on the surface of the graphene flakes. In the MoS_2_/rGO___600 sample, approximately 14.6 wt% of MoS_2_ is present in the volume.

**Figure 4:**
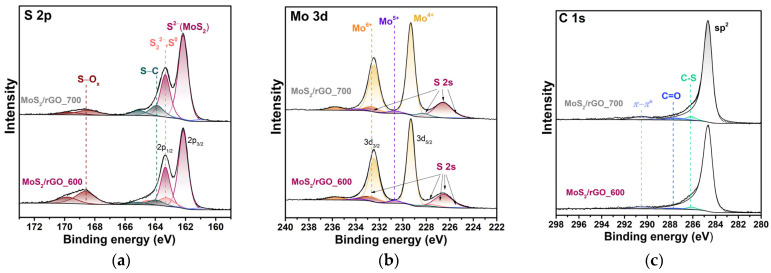
XPS (**a**) S 2p, (**b**) Mo 3d, and (**c**) C 1s spectra of the hybrid materials MoS_2_/rGO_600 (bottom) and MoS_2_/rGO_700 (top).

The XPS S 2p spectra of the hybrids fitted by spin–orbit doublets are compared in Figure 4a. The intense doublet with a S 2p_3/2_ energy of ~162.2 eV corresponds to S^2−^ in MoS_2_ [38]. The S 2p_3/2_ position at ~163.2 eV is assigned to the edge dimers S_2_^2−^ or to elemental sulfur S_0_ [39]. This doublet is present only in the spectrum of the sample synthesized at 600 °C, which is consistent with the higher Mo to S ratio determined from the survey XPS spectra. A doublet with the S 2p_3/2_ component at 163.9 eV could be assigned to the S–C bonds at the MoS_2_/rGO interface [40,41]. The intensity of this component increases with the increasing synthesis temperature, which is consistent with the appearance of vertically oriented layers in MoS_2_ observed in the TEM images (Figure 2). This component is formed during the rapid heating of the material under conditions of the high local pressure of the released gases (H_2_S and NH_3_), and has been demonstrated in our earlier work [40]. The higher energy doublet with S 2p_3/2_ at ~168.3–168.4 eV indicates the presence of oxidized sulfur states on the sample surface [42]. A low-energy shoulder is due to the MoS_2_ edge-like sites [43]. These states may be responsible for bonding the Mo edges with the oxygen groups present in rGO [40].

Sulfur states were taken into account when fitting the Mo 3d spectrum, which partially overlaps the S 2s binding energy region (Figure 4b). The S 2s peaks at 226.5, 227.6, 228.3, and 232.8 eV are contributed by the components corresponding to the S^2−^, S_2_^2−^, S–C, and SO_x_ states. The high-resolution XPS spectra of Mo 3d (Figure 4b) were fitted by three spin–orbit doublets. The dominant low-energy doublet with a Mo 3d_5/2_ binding energy of 229.3 eV is assigned to the Mo^4+^ state in MoS_2_ [38]. The presence of a high-energy peak indicates molybdenum in an oxidation state higher than +4. The Mo 3d_5/2_ binding energy of 232.7 eV corresponds to Mo^6+^ states [44], which can be formed during the storage of samples under laboratory conditions. To correctly reproduce the spectral intensities, an additional doublet (Mo 3d_5/2_ at ~230.7 eV) assigned to the Mo^5+^ states was added [39].

The XPS C 1s spectra of the samples exhibit a dominant asymmetric peak at ca. 284.6 eV (Figure 4c), which is characteristic of sp^2^-hybridized carbon [45]. The high intensity of the peak indicates the high level of GO reduction. The weak component at 287.7 eV is attributed to carbon directly bonded to oxygen in carbonyl groups [45]. The high-energy component at 290.5 eV represents a shake-up feature associated with the π–π* transitions [46,47]. Moreover, we identified a shoulder at ca. 286.1 eV from the C–S bonding between rGO and MoS_2_ in the hybrid [40], which is consistent with the S 2p spectra.

Based on the presented data, we conclude that the simultaneous thermolysis of GO and (NH_4_)_2_MoS_4_ at temperatures of 600 or 700 °C leads to the formation of a MoS_2_/rGO hybrid nanomaterial with a different orientation of the MoS_2_ layers over the entire surface of the thin graphene layers. According to the Raman spectroscopy and XRD data, the MoS_2_ layers have a hexagonal structure with slightly increased interlayer distance. The XPS data demonstrated the formation of tight contacts between components in the hybrids.

### 3.2. Electrochemical Properties

**Figure 5:**
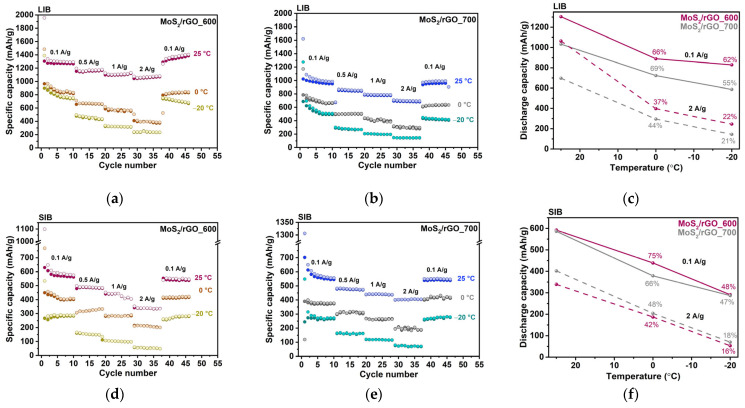
Temperature dependence of the electrochemical performance of the MoS_2_/rGO_600 and MoS_2_/rGO_700 anodes in LIBs (top) and SIBs (bottom). Rate capability of (**a**,**d**) MoS_2_/rGO_600 and (**b**,**e**) MoS_2_/rGO_700 at current densities ranging from 0.1 A/g to 2 A/g and at temperatures 25 °C, 0 °C, and −20 °C. (**c**,**f**) The retention of capacity as the temperature drops from 25 °C to −20 °C at current densities of 0.1 and 2 A/g.

The electrochemical performance of the hybrid anode materials was evaluated at RT and LT (0 and −20 °C). The rate performances at 25 °C, 0 °C, and −20 °C are shown in Figure 5. We first analyzed the measurements performed at RT to establish the relationship between the morphology, structure, and electrochemical behavior of the material. The theoretical capacity of the hybrids, calculated based on the mass ratio of the components determined by TG analysis and the theoretical capacities of MoS_2_ and graphite, is 574 mAh/g for MoS_2_/rGO_600 and 545 mAh/g for MoS_2_/rGO_700. In the first cycle at a current density of 0.1 A/g, the discharge capacities are 1956 and 1621 mAh/g for MoS_2_/rGO_600 and MoS_2_/rGO_700, respectively, in the LIBs; and 1098 and 1306 for MoS_2_/rGO_600 and MoS_2_/rGO_700, respectively, in the SIBs. The initial specific charge capacities for MoS_2_/rGO_600 and MoS_2_/rGO_700 are 1305 and 1022 mAh/g, respectively, in the LIBs; and 629 and 701 mAh/g, respectively, in the SIBs. The lower capacities in the SIBs are explained by the limited interlayer distance for large Na^+^ ions and slower ion diffusion.

The initial Coulombic efficiency (CE) is quite satisfactory and amounts to 54–67%. After 10 cycles at the same current density, the CE is 97%. The higher irreversible capacity in the first cycles is explained by the formation of a SEI film on the surface of the electrode material and the degradation of the electrolyte [48]. In later cycles, the charge–discharge curves almost completely overlap, indicating the stability of the electrodes and the SEI film.

Both electrodes show outstanding cycling and rate performance in LIBs and SIBs at RT. In the LIBs, the reversible specific capacities of MoS_2_/rGO_600 are 1300, 1169, 1105, and 1074 mAh/g at current densities of 0.1, 0.5, 1, and 2 A/g, respectively (Figure 5a). The corresponding capacity values in the SIBs are 593, 490, 443, and 338 mAh/g (Figure 5d). The sample synthesized at a higher temperature of 700 °C has slightly lower LIB capacity values, from 1018 to 695 mAh/g with an increase in current density from 0.1 to 2 A/g (Figure 5b). The SIB capacities of MoS_2_/rGO_700 are 575 mAh/g at 0.1 A/g and 405 mAh/g at 2 A/g (Figure 5e). The values are comparable to the corresponding values for the MoS_2_/rGO_600 electrode at lower current densities (0.1–1 A/g) and become higher at higher current density (2 A/g). In the LIB case, the higher capacity of MoS_2_/rGO_600 is due to the higher content of sulfur, as observed from the XPS S 2p spectrum (Figure 4a). Sulfur species reversibly interact with Li^+^ ions, which contributes to the overall capacity of the electrode (Figures S4 and S5, Tables S1 and S2). In the SIBs, differences in the sulfur content do not have a significant effect on the specific capacities, which is probably due to the limited diffusion of Na^+^ ions into the interface, where the sulfur species are located. The contribution of the rGO component to the hybrid capacity was estimated from the analysis of the charging curves measured at a current density of 0.1 A/g at the 45^th^ cycle of cell operation (Table S2). In the LIBs, the reduction in the contributed capacity from 259 mAh/g for MoS_2_/rGO_600 to 205 mAh/g for MoS_2_/rGO_700 is due to the thinner graphene stacks in the latter case, which provide less interlayer space in rGO for lithium accommodation. The contribution of the rGO component to the capacity in SIBs ranges from 7 mAh/g for MoS_2_/rGO_600 to 12 mAh/g for MoS_2_/rGO_700. Na^+^ ions mainly interact with the surface of the graphene layers and the small contribution from this interaction to the electrochemical capacity indicates that almost the entire rGO surface is covered by MoS_2_ in the hybrids. The reversible specific capacity measured for the control rGO600 sample at 0.1 A/g is 178 mAh/g in the LIB and 128 mAh/g in the SIB (Figure S6). These values differ from those determined for the MoS_2_/rGO_600 sample, confirming that the simultaneous thermolysis of ATM and GO affects the structure of the resulting rGO.

The capacity decreases with increasing current density due to the ion diffusion limitations during charge storage. The highest capacity retention of ~83% with an increase in current density from 0.1 to 2 A/g is observed for the MoS_2_/rGO_600 hybrid material at RT in the LIBs (Figure 5a). This is explained by its unique morphology formed by sandwiching MoS_2_ between graphene layers (C/MoS_2_/C). The decrease in capacity retention to ~57% in the SIBs (Figure 5d) is consistent with the slower diffusion of Na^+^-ions. For the MoS_2_/rGO_700 sample, the capacity retention is ~70% in both the LIBs and SIBs. The same diffusion rate of Li^+^ ions and Na^+^ ions could be expected in the space of the vertically oriented MoS_2_ layers fixed on the graphene surface in this sample.

Experiments with LIBs and SIBs at 0 and −20 °C showed that both hybrid materials can withstand an applied current density of 2 A/g (Figure 5a,b,d,e). When the temperature decreases from 25 °C to −20 °C, the MoS_2_/rGO_600 electrode retains 62% of its capacity at 0.1 A/g and 22% at 2.0 A/g in the LIBs (Figure 5c). The corresponding values decrease to 48% and 16% in the SIBs (Figure 5f). For the MoS_2_/rGO_700 electrode in the LIBs (SIBs), the capacity retention with decreasing temperature is 55% (47%) at 0.1 A/g and 21% (18%) at 2 A/g. Thus, the MoS_2_/rGO_600 and MoS_2_/rGO_700 electrodes have a similar temperature drop resistance in the SIBs, while the MoS_2_/rGO_600 electrode outperforms the MoS_2_/rGO_700 electrode in the LIBs at lower current densities.

At the lowest temperature of −20 °C and the highest current density of 2 A/g, the samples have good reversible discharge capacities of 245 mAh/g for MoS_2_/rGO_600 and 145 mAh/g for MoS_2_/rGO_700 in the LIBs, and 53 mAh/g for MoS_2_/rGO_600 and 70 mAh/g for MoS_2_/rGO_700 in the SIBs.

**Figure 6:**
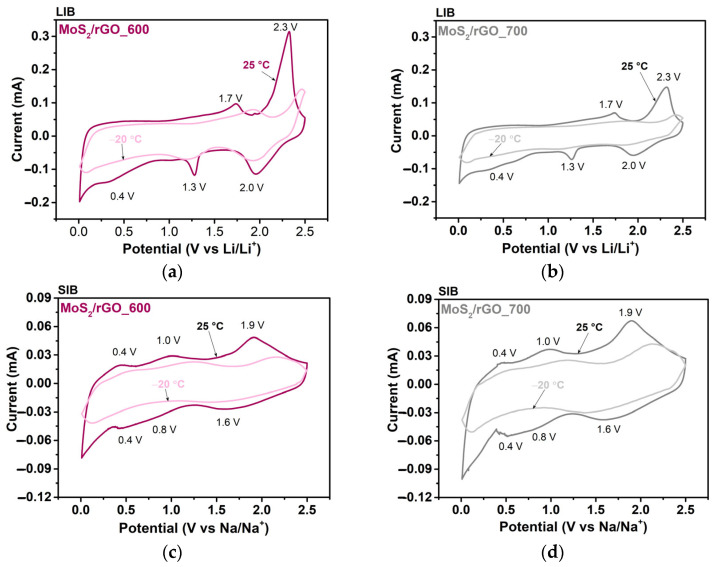
CV curves taken on cycle 3 of (**a**,**c**) MoS_2_/rGO_600 and (**b**,**d**) MoS_2_/rGO_700 in (**a**,**b**) LIBs and (**c**,**d**) SIBs measured at a scan rate of 0.1 mV/s at RT (25 °C) or LT (−20 °C).

The electrochemical processes in the batteries were analyzed using CV. Figure 6 compares the CV curves measured at 25 °C and −20 °C. The anodic peak at 2.3 V vs. Li/Li^+^ and the corresponding cathodic peak at 2.0 V vs. Li/Li^+^ observed for the LIB cells at 25 °C (Figure 6a,b) correspond to the interaction of Li^+^ ions with sulfur. The height of this peak is reduced in the MoS_2_/rGO_700 sample, which can be explained by the lower sulfur content according to the XPS data. The redox pair at 1.7/1.3 V vs. Li/Li^+^ is attributed to the deintercalation/intercalation of Li^+^ ions from/into MoS_2_. During the intercalation process, Li^+^ ions are introduced into the interlayer space, and as the amount of introduced Li^+^ increases, a phase transition from 2H to 1T-Li_x_MoS_2_ occurs [49]. The broad peak at ~0.4 V vs. Li/Li^+^ indicates a long conversion process, i.e., the transition of Li_x_MoS_2_ to Mo and Li_2_S. A peak at the potential below 0.2 V vs. Li/Li^+^ corresponds to the intercalation of Li^+^ ions into the interlayer space of the rGO layers. The contribution of the carbon component to the capacity is estimated at approximately 20% (Table S2). As the electrochemical test temperature decreases to −20 °C, the height of the redox peaks decreases, indicating a slowdown in the redox processes and leading to a deterioration in the battery performance. The observed shift of the anodic peaks towards higher potential values indicates an increase in energy consumption for the reactions.

The CV curves measured for the hybrids in SIBs are shown in Figure 6c,d. The CV curves exhibit three pairs of reversible redox peaks at potentials of 1.9/1.6, 1.0/0.8, and 0.4/0.4 V vs. Na/Na^+^. The first pair corresponds to the interaction of Na^+^ ions with sulfur, the second is due to the intercalation of Na^+^ between MoS_2_ layers, and the third pair indicates the reversible conversion of Na_x_MoS_2_ to Mo and Na_2_S (Tables S1 and S2) [50,51,52]. A peak below the potential of 0.2 V vs. Na/Na^+^ is related to the electrochemical interaction of Na^+^ ions with rGO. When the measurement temperature is reduced to −20 °C, the electrochemical reactions of the SIBs are practically absent, which explains the rapid drop in specific capacity with increasing current density (Figure 5b,e).

**Figure 7:**
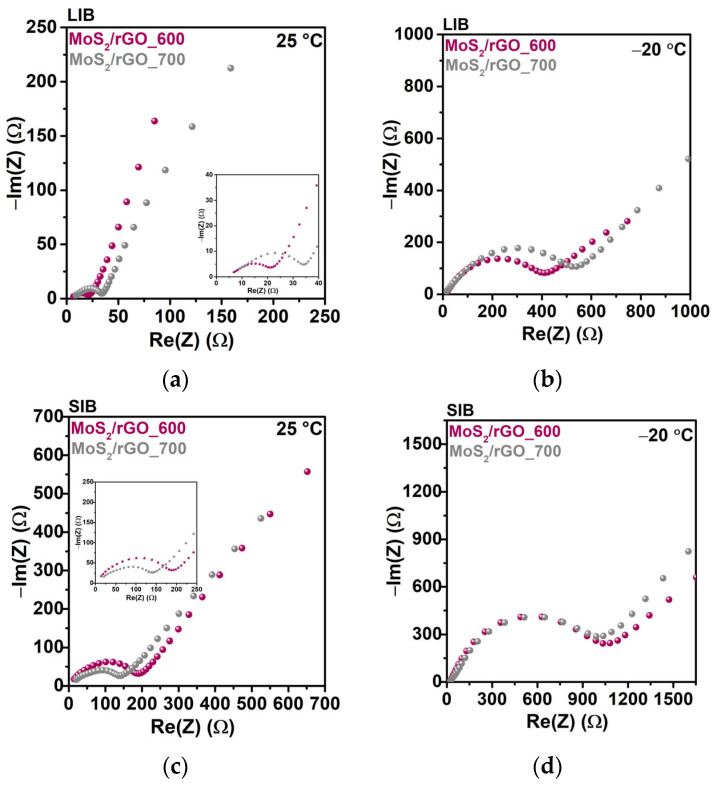
Nyquist plots of the EIS of MoS_2_/rGO_600 and MoS_2_/rGO_700 in (**a**,**b**) LIBs and (**c**,**d**) SIBs at operation temperatures of (**a**,**c**) 25 °C and (**b**,**d**) −20 °C.

The temperature dependence of the electrochemical impedance spectra of the LIB cells with the MoS_2_/rGO_600 and MoS_2_/rGO_700 electrodes at two operation temperatures of 25 and −20 °C was investigated to reveal the charge-transfer processes for the electrodes and SEI. EIS spectra were measured at a full state of charge (2.5 V vs. Li/Li^+^ or Na/Na^+^) in the fixed frequency range from 10 kHz to 0.05 Hz. The Nyquist plots (Figure 7) are composed of two regions: the semicircle in the middle-to-high frequency corresponds to the interfacial impedance and the charge-transfer resistance, while the straight line in the low frequency relates to the diffusion of Li^+^ or Na^+^ in the electrode material. The semicircle magnitude ends at 21 Ohm for MoS_2_/rGO_600 and 34 Ohm for MoS_2_/rGO_700 in the LIBs at RT (Figure 7a), and increases to 410 Ohm and 540 Ohm at −20 °C, respectively (Figure 7b). The surface charge-transfer resistance of MoS_2_/rGO_600 is lower than that of MoS_2_/rGO_700, indicating better Li^+^ ion transport in the former sample. When the temperature decreases to −20 °C, the semicircle in the high-frequency region becomes larger in a similar way for the EIS profiles of both electrodes. Both the SEI and charge-transfer resistances increase when the temperature drops, which is in the good accordance with the Arrhenius law for the semiconducting behavior of conductivity.

The electrode resistance values in the SIBs (Figure 7c,d) are elevated due to the more challenging diffusion of larger Na^+^ ions. LT has an obvious limiting effect on the charge-transfer process. In comparison, the high and mid-frequency resistances for cells cycled at −20 °C are approximately five times higher than those for cells cycled at 25 °C, showing that abundant SEI is formed at low temperatures, resulting in slow Na^+^ ion transport.

**Figure 8:**
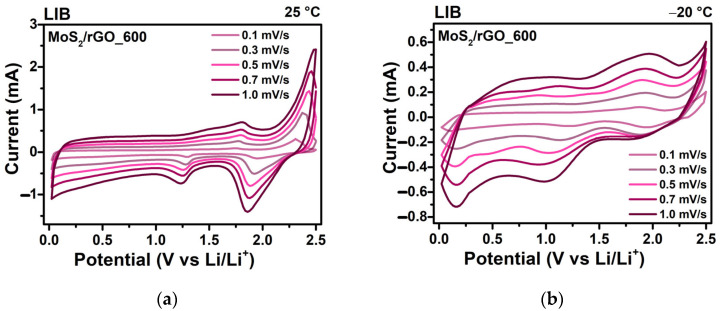

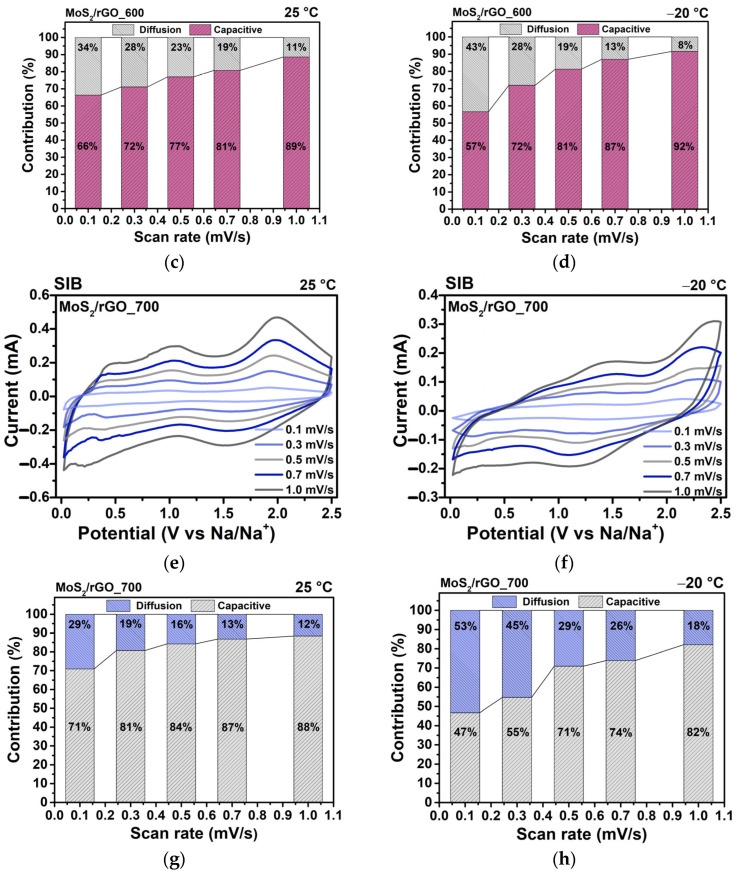
CV curves of (**a**,**b**) MoS_2_/rGO_600 in LIBs and (**e**,**f**) MoS_2_/rGO_700 in SIBs at operation temperatures of (**a**,**e**) 25 °C and (**b**,**f**) −20 °C and at scan rates ranging from 0.1 to 1.0 mV/s. Contribution of surface-controlled capacitive and diffusion-controlled reactions at various scan rates for (**c**,**d**) MoS_2_/rGO_600 in LIBs and (**g**,**h**) MoS_2_/rGO_700 in SIBs.

To study the charge accumulation kinetics of the electrode materials, a temperature- and rate-dependent CV test was conducted. CV curves were measured after the temperature-dependent GDC test at 25 °C and then at −20 °C. The rectangular profile of the CV curves (Figure 8a,b,e,f) corresponds to the adsorption of solvated Li^+^/Na^+^ ions forming an electrical double layer on the electrode surface. The peaks observed in the CV curves are attributed to the redox reactions of desolvated Li^+^/Na^+^ ions inside the electrode. The reduction in the operation temperature results in a decrease in the height of the redox peaks, as well as in the current response of the cells. The total current response (*i*) can be represented as a function of the scan rate (*v*) using the following formula: *i* = *k*_1_*v* + *k*_2_*v*^½^, as the sum of the contributions from the surface-controlled capacitive current (*k*_1_*v*) and the diffusion-limited current (*k*_2_*v*^½^) [53]. The values of *k*_1_ and *k*_2_ are determined from the slope and intercept of the experimental plots of *i*/*v*^½^ vs. *v*^½.^ For both the LIBs and SIBs electrodes, capacitive charge storage and surface redox reactions play a dominant role (Figure 8c,g), and these surface-controlled processes at the electrode increase with the increasing scan rate. The increase in the capacitive contribution observed for MoS_2_/rGO_600 in the LIB with a decreasing operation temperature from 25 °C (Figure 8c) to −20 °C (Figure 8d) is due to the slowing down of the diffusion processes. These include the solid-state diffusion of Li^+^ ions in the electrodes, the transport of solvated Li^+^ ions through the electrolyte, and the charge transfer at the interfaces. In contrast, in the MoS_2_/rGO_700 anode tested in SIBs, the contribution of the diffusion processes increases with a decrease in the operation temperature from 25 °C (Figure 8g) to −20 °C (Figure 8h). It can be assumed that the slow migration of solvated large Na^+^ ions in the viscous electrolyte to the electrode surface kinetically limits the rates of surface electrochemical reactions.

**Figure 9:**
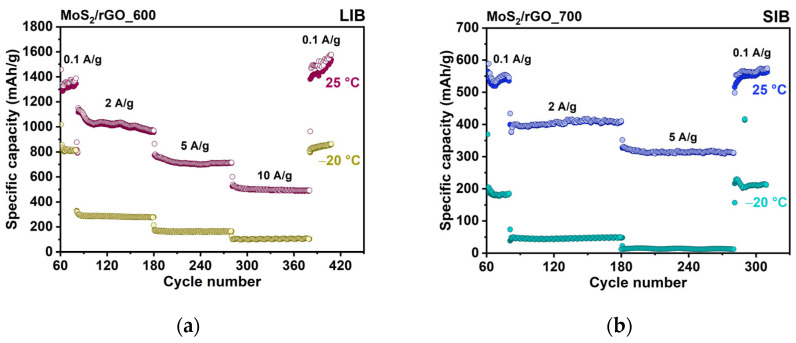
Cycle performance after 60 cycles of (**a**) MoS_2_/rGO_600 at current densities ranging from 0.1 A/g to 10 A/g in LIBs and (**b**) MoS_2_/rGO_700 at current densities ranging from 0.1 A/g to 5 A/g in SIBs at operation temperatures of 25 °C and −20 °C.

After 60 repeated discharges and charges of MoS_2_/rGO_600 and MoS_2_/rGO_700 at operation temperatures of 25 °C or −20 °C, long-term tests were carried out at higher current densities. At current densities of 2, 5, and 10 A/g, the LIB cells with the MoS_2_/rGO_600 electrode provided 1300, 706, and 502 mAh/g, respectively, at 25 °C; and 287, 157, and 103 mAh/g, respectively, at −20 °C (Figure 9a). In the SIBs, the MoS_2_/rGO_700 electrode delivered 410 and 314 mAh/g at RT; and 45 and 14 mAh/g at LT at current densities of 2 and 5 A/g, respectively (Figure 9b). Although both electrodes show stable performance with high Coulombic efficiency (~100%) at both temperatures, a significant decrease in specific capacity is observed at higher current rates at −20 °C. Such electrochemical behavior was attributed to the formation of a lithium coating on the anode [54,55]. However, in our case, upon repeated cycling at 0.1 A/g at −20 °C, the specific capacity was restored in both LIBs and SIBs. Therefore, we excluded the formation of metallic Li or Na particles. The low specific capacity at high current rates and low temperatures may be due to the slow kinetics of charge accumulation.

## 4. Discussion

**Figure 10:**
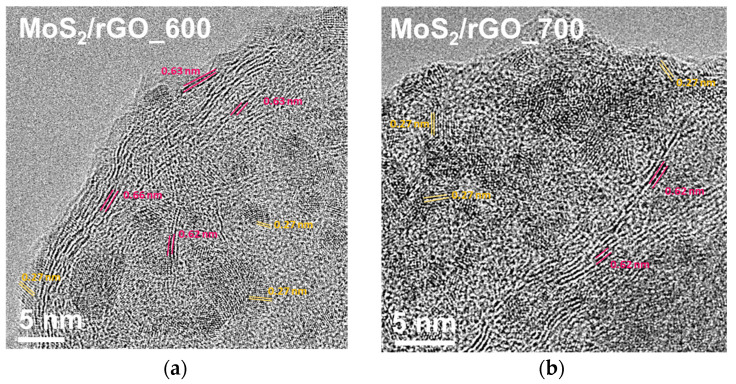
TEM images of (**a**) MoS_2_/rGO_600 anode after 410 cycles in LIB and (**b**) MoS_2_/rGO_700 anode after 310 cycles in SIB.

Electrochemical studies of the MoS_2_/rGO hybrids synthesized by the rapid decomposition of ATM/GO aerogel at 600 and 700 °C showed their highly stable performance in LIBs and SIBs under RT and LT conditions. After long-term tests of 410 cycles in the LIB and 310 cycles in the SIB at different current densities, the coin cells were disassembled and the anode materials on the copper substrates were washed in DEC solvent to remove the electrolyte. TEM images of the MoS_2_/rGO_600 and MoS_2_/rGO_700 electrodes showed the preservation of the MoS_2_ layers (some of them are highlighted in pink in Figure 10). The interlayer spacing is about 0.62–0.66 nm. Nanocrystals of 1.6–4 nm in size are also observed on the surface of the samples. The distance between the fringes of 0.27 nm (highlighted in orange in Figure 10) corresponds to the separation of the (100) planes in MoS_2_ [56].

The XPS survey spectra of the MoS_2_/rGO_600 and MoS_2_/rGO_700 electrodes after cycling in the LIBs or SIBs, respectively, revealed the presence of C, F, O, and Li or Na as dominant elements and traces of Cl and S (Figure S7a). The spectra did not detect signals from molybdenum since the electrode surface is covered with the SEI layer. However, the XPS S 2p spectra of the samples were measured, which showed an increase in the oxidized sulfur forms (peak at ~168.5 eV) and a broadening of the main peak at ~162 eV (Figure S7b) as compared to the pristine MoS_2_/rGO samples (Figure 4a). The peak broadening is due to the different forms of sulfur, such as the S^2–^ states in 2H-MoS_2_, 1T-MoS_2_, and Li_2_S/Na_2_S. The peaks in the S 2p spectrum of MoS_2_/rGO_600 are broader than those of MoS_2_/rGO_700, which may be related to the greater change in the structure of the MoS_2_ component in the LIB than in the SIB.

The analysis of the CDG data (Tables S1 and S2) revealed that the mechanisms of charge accumulation in MoS_2_ are similar for LIBs and SIBs. They involve the intercalation of Li^+^ ions into MoS_2_ and the conversion of the intercalate into Mo and Li_2_S/Na_2_S. According to the CV data (Figure 6), this conversion reaction is reversible, and the TEM study of the electrodes after long-term cycling (Figure 10) confirm this. The preservation of the layered structure of MoS_2_ is attributed to strong bonding between MoS_2_ and rGO, as shown by XPS (Figure 4a). The interaction of alkali metal ions with excess sulfur also makes a significant contribution to the capacity of the hybrids (Tables S1 and S2). The main difference in the electrochemical behavior of MoS_2_/rGO in the LIBs and SIBs is due to the rGO component. Li^+^ ions are easily intercalated into the space between graphitic layers, whereas Na^+^ ions are not. As a result, rGO contributes more to the capacity of LIBs, while in the case of SIBs, its main role is to stabilize MoS_2_.

The results obtained in this work are compared with the reported data on the electrochemical performance of composites in LIBs or SIBs under LT conditions in Table 2.

The specific capacities provided by the MoS_2_/rGO_600 electrode are among the best values. At the selected current densities of 0.1, 0.5, 1, and 2 A/g, this electrode is only inferior to one material from the list, namely, the MoS_2_/C composite, where an increased interlayer distance was observed [26]. The capacities of MoS_2_/G materials do not exceed the values obtained in this work. Other materials do not contain MoS_2_ and demonstrate poorer capacity characteristics, which once again emphasizes the prospects of the selected material. In SIBs, characteristics comparable to our results were obtained at low current densities in two works [61,62] and surpass ours with increasing current density. However, both materials [61,62] have a complex composition and a lengthy synthesis procedure, while the approach we propose is fast and scalable.

**Figure 11:**
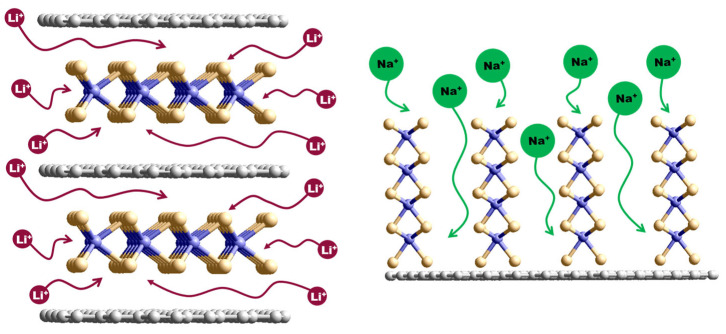
Schematic illustration of the interaction of MoS_2_/rGO hybrid materials with Li^+^ ions (left model) and Na^+^ ions (right model). Gray, brown, and violet balls correspond to carbon, sulfur, and molybdenum, respectively.

The electrochemical reactions occurring during the interaction of MoS_2_ with Li^+^ and Na^+^ ions are similar, but their contribution to the total capacity differs and depends on the relative orientation of the MoS_2_ and rGO layers. Figure 11 schematically shows the difference in the structure of the MoS_2_/rGO_600 and MoS_2_/rGO_700 hybrids, which determines the preference of the anodes for LIBs and SIBs, respectively. The rapid thermolysis of ATM/GO aerogel at 600 °C results in a predominantly horizontal alignment of graphene and MoS_2_ layers (left model in Figure 11) in the hybrid. This unique architecture gives it outstanding electrochemical properties as a LIB anode material [65]. The close contact between the MoS_2_ and the graphitic layer enhances the electrode conductivity, but also improves the reversibility of the conversion reaction, preventing the oxidation of Li_2_S to polysulfides. Meanwhile, the outer carbon layers protect the MoS_2_ layer, providing the electrode with high structural stability [66].

Increasing the synthesis temperature to 700 °C promotes the vertical attachment of the MoS_2_ layers to the rGO surface (right model in Figure 11). As shown in [67], the vertical orientation of MoS_2_ layers leads to the formation of nanowalls. These nanowalls increase the contact area between the electrode material and the electrolyte, thereby reducing the diffusion pathways of Na^+^ ions in SIBs. It was reported that Na^+^ ions can be deintercalated more efficiently and rapidly along the vertical direction of the nanowalls. In addition, the formation of C–S bonds enhances the interaction between MoS_2_ and rGO, improving structural stability and electron transport. A key factor is the coating of the carbon surface with MoS_2_, since this reduces the direct interaction area between the carbon and the electrolyte. This reduces undesirable capacity loss due to the irreversible loss of Na^+^ ions [68].

## 5. Conclusions

In summary, we synthesized MoS_2_/rGO hybrids by the rapid decomposition of ATM/GO aerogel at 600 and 700 °C and found their high capacity and rate performance as LIB and SIB anodes at RT and LT. Both materials showed similar characteristics in terms of morphology (detected by SEM) and defect density (detected by Raman spectroscopy). XPS revealed a higher sulfur content in the MoS_2_/rGO_600 hybrid. TEM data demonstrated that synthesis at 600 °C resulted in the formation of structures composed of aligned rGO and MoS_2_ layers. The MoS_2_/rGO_700 sample contained more vertically oriented MoS_2_ layers with respect to the graphene surface. The MoS_2_/rGO_600 sample showed an excellent reversible capacity of 1400 mAh/g at a current density of 0.1 A/g in the LIBs at RT and a remarkable rate performance (1074 mAh/g at 2 A/g). Reducing the operation temperature to −20 °C allowed up to 62% of the capacity to be retained at 0.1 A/g. The influence of the layer orientation and the interface was less significant in the SIBs, resulting in the good performance of both hybrids. The SIB values for both materials reached 580 mAh/g at 0.1 A/g and 400 mAh/g at 2 A/g, while reducing the temperature to −20 °C retained 48–17% of the capacity, respectively. The decrease in specific capacity at LTs was attributed to the increase in SEI and charge-transfer resistances, as well as the deterioration of Li^+^ or Na^+^ ion diffusion. The predominant surface-controlled capacitive behavior of charge storage in the MoS_2_/rGO hybrids explained the high capacity retention at LT conditions in LIBs and SIBs.

## Figures and Tables

**Table 1:** Content of elements in the MoS_2_/rGO hybrids expected from decomposition reactions and determined by the analysis of XPS and TG data.

Sample	Expected, wt%			XPS, wt%				TG, wt%	
	C	S	Mo	C	S	Mo	O	C	Mo + S
MoS_2_/rGO_600	25	30	45	33.2	26.3	31.4	9.1	31.6	68.4
MoS_2_/rGO_700	25	30	45	37.0	23.6	32.1	7.3	41.9	58.1

**Table 2:** Comparison of the performance of MoS_2_/rGO materials in LIBs and SIBs under LT conditions with the examples of electrodes reported in the literature. Values exceeding capacities obtained in this work are highlighted in bold.

	Sample	Temperature, °C	Specific Capacity (mAh/g) at Given Current Density (A/g)				Ref.
			0.1	0.5	1	2	
**LIB**	MoS_2_/rGO_600	−20	**763**	**458**	**322**	**245**	this work
	MoS_2_/rGO_700	−20	510	279	200	144	this work
	MoS_2_/C	−20	**775**	**583.2**	**491.8**	**342.8**	[26]
	MoS_2_/G	−20	~700	~360	−	−	[25]
	MnO@Graphite	−25	210	26	−	−	[57]
	G/NTO-4 (TiO_2_@TiN/graphene-400 °C)	−20	265	202	185	166	[58]
	FeS@g-C	−20	624.3 (0.05 A/g)	367.1	246.7	104.9	[59]
	SnSe@C	−20	420	−	−	−	[60]
**SIB**	MoS_2_/rGO_600	−20	290	151	100	53	this work
	MoS_2_/rGO_700	−20	272	163	118	76	this work
	WS_2_/MoS_2_/Ti_3_C_2_T_x_ MXene	−20	**293.7**	**229.2**	**182.5**	**107.8 (3A/g)**	[61]
	ZnSe@NCNFs	−20	**305.3** **(0.02 A/g)**	**264**	**236.7**	**181.8**	[62]
	LS-Sb@G	−20	226.2	−	−	−	[63]
	a-KTiO_x_/Ti_2_CT_x_	−25	124.9	90.7	−	70.4	[64]
	µm-MoS_2_	−30	213.8 (0.05 A/g)	−	−	−	[24]

## Data Availability

The data that support the findings of this study are available on request from the corresponding author.

## Supplementary Materials

The following supporting information can be downloaded at: https://www.mdpi.com/article/10.3390/nano15110824/s1, Figure S1: Low−resolution TEM images of hybrid materials; Figure S2: XRD patterns of hybrid materials; Figure S3: TG analysis of hybrid materials; Figure S4: GDC curves at 2^nd^ operation cycle measured at 25 °C; Table S1: Contributions of electrochemical processes to the capacity of electrodes at 2^nd^ operation cycle; Figure S5: GDC curves at 45^th^ operation cycle measured at 25 °C; Table S2: Contributions of electrochemical processes to the capacity of electrodes at 45^th^ operation cycle. Figure S6: Rate capability of rGO600 in LIBs and SIBs measured at 25 °C. Figure S7: XPS survey and S 2p spectra of hybrid electrodes after long-term cycling.
