# Supplementary material for: Molybdenum Disulfide and Reduced Graphene Oxide Hybrids as Anodes for Low-Temperature Lithium- and Sodium-Ion Batteries
Source: Nanomaterials (Basel). 2025 May 29;15(11):824. doi: 10.3390/nano15110824 (PMC12157020; doi:10.3390/nano15110824)
Supplement: Supplementary file 1 [file nanomaterials-15-00824-s001.zip › nanomaterials-3646085-supplementary.pdf]

Supplementary Materials

Molybdenum disulfide and reduced graphene oxide hybrids as anodes for low-temperature lithium- and sodium-ion batteries

Anna A. Vorfolomeeva [1], Alena A. Zaguzina [1], Evgeny A. Maksimovskiy [1], Artem V. Gusel'nikov, Pavel E. Plyusnin, Alexander V. Okotrub [1], Lyubov G. Bulusheva [1,*]

[1] Nikolaev Institute of Inorganic Chemistry SB RAS, 3 Acad. Lavrentiev ave., 630090, Novosibirsk, Russia; vorfolomeeva@niic.nsc.ru (A.A.V.); kotsun@niic.nsc.ru (A.A.Z.); eugene@niic.nsc.ru (E.A.M.); gusel@niic.nsc.ru (A.V.G.); plus@niic.nsc.ru (P.E.P); spectrum@niic.nsc.ru (A.V.O.)

* Correspondence: bul@niic.nsc.ru (L.G.B.)

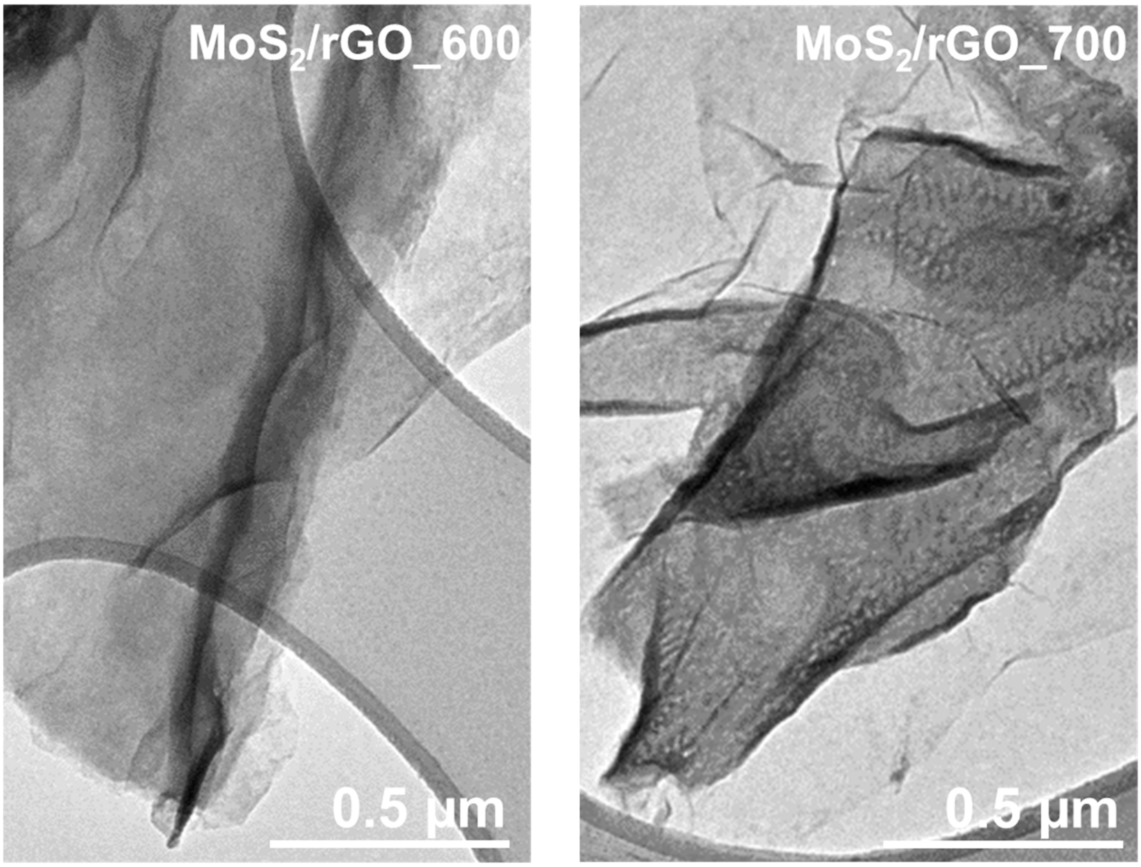

(**a**) (**b**)

Figure S1. Low−resolution TEM images of (**a**) MoS_2/rGO_600 and (**b**) MoS_2/rGO_700.

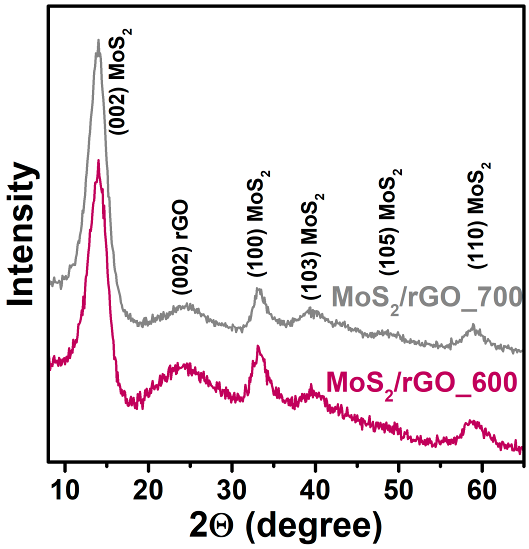

Figure S2. XRD patterns of MoS_2/rGO hybrid materials.

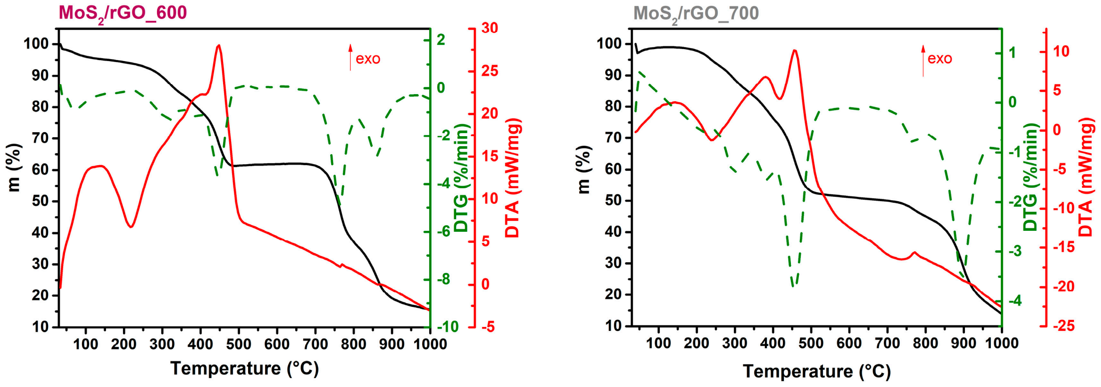

(**a**) (**b**)

Figure S3. TG analysis (**a**) MoS_2/rGO_600 and (**b**) MoS_2/rGO_700.

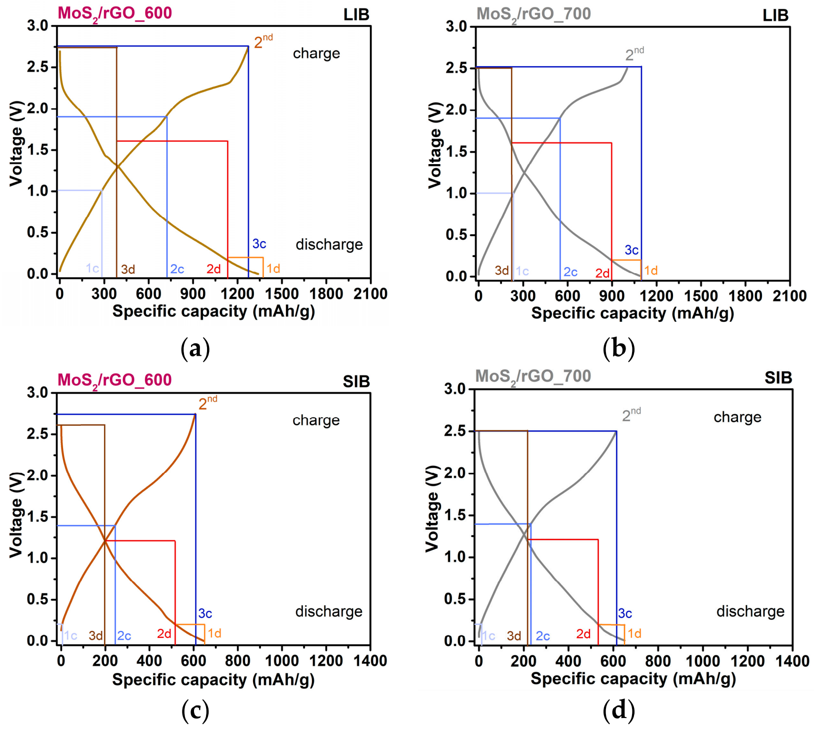

Figure S4. GDC curves at 2nd operation cycle measured at 25 °C for (**a, c**) MoS_2/rGO_600 and (**b, d**) MoS_2/rGO_700 in (**a, b**) LIBs and (**c, d**) SIBs with reactions' contribution: (1) $xM^+ + C + xe^- \rightarrow M_xC$, (2) $MoS_2 + 4M^+ + 4e^- \rightarrow Mo + 2M_2S$, (3) $nS + 2M^+ + 2e^- \rightarrow M_2S_n$

Table S1. Contributions of electrochemical processes (1), (2) and (3) to the capacity of 600_3:1 and 700_3:1 electrode at 2nd operation cycle in LIBs and SIBs.

M	Sample	process	(1) $xM^+ + C + xe^- \rightarrow M_xC$	(2) $MoS_2 + 4M^+ + 4e^- \rightarrow Mo + 2M_2S$	(3) $nS + 2M^+ + 2e^- \rightarrow M_2S_n$
Li	MoS_2/rGO_600	discharge	209 mAh/g (~15.6%)	749 mAh/g (~55.9%)	381 mAh/g (~28.5%)
		charge	285 mAh/g (~22.4%)	433 mAh/g (~34.0%)	556 mAh/g (~43.6%)
	MoS_2/rGO_700	discharge	216 mAh/g (~19.9%)	681 mAh/g (~62.6%)	190 mAh/g (~17.5%)
		charge	234 mAh/g (~23.4%)	313 mAh/g (~31.3%)	454 mAh/g (~45.3%)
Na	MoS_2/rGO_600	discharge	199 mAh/g (~30.8%)	320 mAh/g (~49.4%)	128 mAh/g (~19.8%)
		charge	6 mAh/g (~1%)	239 mAh/g (~39.4%)	362 mAh/g (~59.6%)
	MoS_2/rGO_700	discharge	214 mAh/g (~32.9%)	317 mAh/g (~48.8%)	119 mAh/g (~18.3%)
		charge	9 mAh/g (~1.5%)	222 mAh/g (~36.2%)	382 mAh/g (~62.3%)

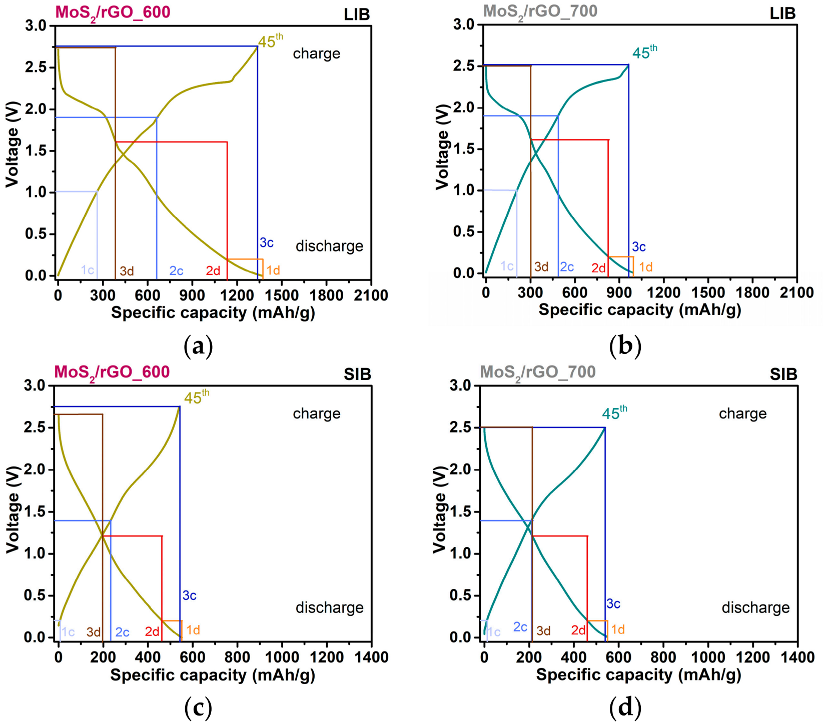

Figure S5. GDC curves at 45th operation cycle measured at 25 °C for (**a, c**) MoS_2/rGO_600 and (**b, d**) MoS_2/rGO_700 in (**a, b**) LIBs and (**c, d**) SIBs with reactions' contribution: (1) $xM^+ + C + xe^- \rightarrow M_xC$, (2) $MoS_2 + 4M^+ + 4e^- \rightarrow Mo + 2M_2S$, (3) $nS + 2M^+ + 2e^- \rightarrow M_2S_n$

Table S2. Contributions of electrochemical processes (1), (2) and (3) to the capacity of 600_3:1 and 700_3:1 electrode at 45th operation cycle in LIBs and SIBs.

M	Sample	process	(1) $xM^+ + C + xe^- \rightarrow M_xC$	(2) $MoS_2 + 4M^+ + 4e^- \rightarrow Mo + 2M_2S$	(3) $nS + 2M^+ + 2e^- \rightarrow Mi_2S_n$
Li	MoS_2/rGO_600	discharge	233 mAh/g (~17.0%)	758 mAh/g (~55.2%)	381 mAh/g (~27.8%)
		charge	259 mAh/g (~19.3%)	400 mAh/g (~30.0%)	678 mAh/g (~50.7%)
	MoS_2/rGO_700	discharge	170 mAh/g (~17.1%)	522 mAh/g (~52.5%)	303 mAh/g (~30.4%)
		charge	205 mAh/g (~21.3%)	281 mAh/g (~29.2%)	475 mAh/g (~49.5%)
Na	MoS_2/rGO_600	discharge	90 mAh/g (~16.3%)	265 mAh/g (~48.0%)	197 mAh/g (~35.7%)
		charge	7 mAh/g (~1%)	227 mAh/g (~42.0%)	306 mAh/g (~57.0%)
	MoS_2/rGO_700	discharge	90 mAh/g (~16.4%)	247 mAh/g (~44.9%)	213 mAh/g (~38.7%)
		charge	12 mAh/g (~2.2%)	197 mAh/g (~36.5%)	331 mAh/g (~61.3%)

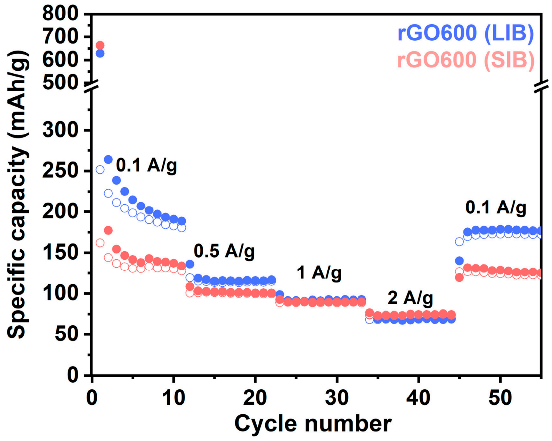

Figure S6. Rate capability of rGO600 in LIBs (blue) and SIBs (red) at current densities ranging from 0.1 A/g to 2 A/g measured at 25 °C.

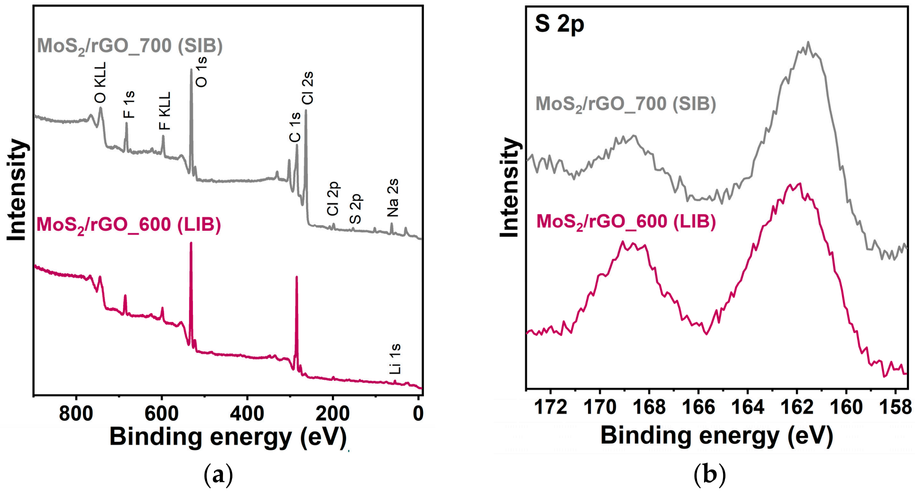

Figure S7. XPS (**a**) survey and (**b**) S 2p spectra of hybrid electrodes MoS_2/rGO_600 (bottom) after 410th discharge-charge cycles in LIBs and MoS_2/rGO_700 (top) after 310th discharge-charge cycles in SIBs.
